# Organosulfurs, *S*-allyl cysteine and *N*-acetyl cysteine sequester di-carbonyls and reduces carbonyl stress in HT22 cells

**DOI:** 10.1038/s41598-023-40291-6

**Published:** 2023-08-11

**Authors:** Reshmee Bhattacharya, Saakshi Saini, Souvik Ghosh, Partha Roy, Nemat Ali, Mohammad Khalid Parvez, Mohammed S. Al-Dosari, Awdhesh Kumar Mishra, Laishram Rajendrakumar Singh

**Affiliations:** 1 Dr. B. R. Ambedkar Center for Biomedical Research, University of Delhi, Delhi, 110007 India; 2 Department of Biosciences and Bioengineering, IIT Roorkee, Roorkee, 247667 Uttarakhand India; 3 Department of Pharmacology and Toxicology, College of Pharmacy, King Saud University, Riyadh, 11451 Saudi Arabia; 4 Department of Pharmacognosy, College of Pharmacy, King Saud University, Riyadh, 11451 Saudi Arabia; 5 Department of Biotechnology, Yeungnam University, Gyeongsan, Gyeongsanbuk-Do Republic of Korea

**Keywords:** Biochemistry, Biophysics, Cell biology, Diseases

## Abstract

Diabetes, characterized by high blood glucose level, is a progressive metabolic disease that leads to serious health complications. One of the major pathological consequences associated with diabetes is the accumulation of highly reactive carbonyl compounds called advanced glycation end products (AGEs). Most of the AGEs are dicarbonyls and have the potential to covalently modify proteins especially at the lysine residues in a non-enzymatic fashion (a process termed as glycation) resulting in the functional impairment and/or toxic gain in function. Therefore, non-toxic small molecules that can inhibit glycation are of interest for the therapeutic intervention of diabetes. In the present communication, we have investigated the effect of organosulfurs (*S*-allyl cysteine, SAC and *N*-acetyl cysteine, NAC) that are major principal components of *Allium sativa* against the glycation of different proteins. We discovered that both SAC and NAC are potent anti-glycating agents. We also found that both SAC and NAC reduce ROS level and inhibit apoptosis caused by protein glycation.

## Introduction

Diabetes is a disease caused due to chronic increase in blood sugar level, a condition called hyperglycemia^1^. Diabetes is associated with various clinical complications including, diabetic neuropathy, nephropathy, retinopathy, cataract, and risk of myocardial infarction etc^2–4^. One of the primary pathological consequences of hyperglycemia are the formation of highly reactive carbonyl compounds called advanced glycation end products (AGEs)^5,6^. The formation of such AGEs initiates with a glycation reaction when the free amino (–NH_2_) group of protein lysine residue attacks the electrophilic carbonyl (–CO) group of reducing sugar in a nucleophilic addition reaction, forming an unstable Schiff’s base, Aldimine. This Schiff’s base further undergoes Amadori rearrangement forming a stable ketoamine compound (Amadori product). The Amadori product then forms 1,2-dicarbonyl compounds (glyoxal, GO; methylglyoxal, MGO) or 2,3-dicarbonyl compounds (1-deoxyglucosone, 1-DG). Additionally, AGEs are formed via oxidative and non-oxidative rearrangements of the Amadori products and subsequent condensation reaction between the dicarbonyls and side chain of lysine, cysteine and arginine residues (e.g., *N*-carboxymethylysine, CML; *N*-carboxyethylysine, CEL, imidazolone derivatives, etc.)^7^. Thus, different dicarbonyls/AGEs are accumulated under diabetic conditions^8^. AGEs are also considered to be 20,000-fold more reactive than their precursor molecules in terms of glycation reaction and therefore, are relatively highly cytotoxic^9^. The process of glycation has been known to consequently alter structure and functional properties of enzymes and/or even results in the formation of high order oligomeric species further exhibiting toxic gain of function^7^. Furthermore, elevated level of such AGEs may interfere with the normal cellular processes because of their interaction with cellular receptors including RAGE and TLRs^10,11^. These consequences are considered to be the main cause of toxicity, increase in oxidative stress, inflammation and apoptosis associated with hyperglycemia. Therefore, proper management and control of AGE production or inhibition of protein adduct formation is clinically important. The existence of various glycated proteins and other AGE-associated cellular toxicities in diabetes has encouraged researchers for identification of molecules that can inhibit glycation. One of the widely used hypoglycemic agents, Metformin is believed to reduce α-dicarbonyl levels and inhibits AGE formation by binding to MGO or 3-deoxyglucosone or by upregulating detoxifying enzymes in vivo^12–14^. Other guanidino compounds have also been known to be anti-glycating agent because of their ability to form complex with AGEs^15,16^. Traditionally, various herbs and plant-based products have been used for the treatment of diabetes^17^. These include *Azadirachta indica*,* Caesalpinioideae*,* Pachira aquatic*,* Gongronema latifolium*,* Allium sativum*,* Carthamus tinctorius*,* Ferula assa-foetida*,* Bauhinia*,* Gymnema sylvestre*, etc^18–21^. Of these, *Allium sativa* commonly called garlic has been shown to be highly effective hypoglycemic plant^22^. One major and most abundant principal component in *Allium sativa* is *S*-allyl cysteine (SAC)^23^. Nowadays, attention has been paid on the use of SAC for different human diseases because of its potent anti-oxidant, anti-inflammatory, anti-arthritis, anti-diabetic and anti-atherogenic properties^24–27^. However, not much is known about its anti-glycation properties. To date, there has been no systematic studies about the use of SAC on this front. In the present manuscript, we have made systematic investigation of the effect of SAC and its analogue NAC, for anti-glycating properties. Using Carbonic anhydrase (CA), we have systematically investigated the effect of SAC/NAC as anti-glycating agent(s) against MGO-induced covalent modification by analyzing various biophysical and biochemical parameters. We have chosen CA because it is a serum protein, elevated in type 2 diabetes and its glycation in vivo has already been reported^28–30^. We have further examined these properties on HT22 cells by analyzing cytotoxicity, ROS levels and apoptosis. We discovered that SAC and NAC are very potent anti-glycating agent. Since NAC is an FDA approved drug for the treatment of triple negative breast cancer, the study implicates that it should be repurposed for the treatment of diabetes.

## Materials and methods

### Materials

Carbonic anhydrase (CA, from bovine erythrocytes), Bovine serum albumin (BSA, from bovine), Transthyretin (TTR, from human plasma), and other chemicals were purchased from Sigma-Aldrich chemical Co. *S*-allyl l-cysteine (SAC) was purchased from Cayman Chemicals. Methylglyoxal (MGO), Glyoxal (GO), Fructose, 8-Anilinonaphthalene-1-sulfonic acid (ANS), Dinitrophenyl hydrazine (DNPH), p-Nitrophenyl acetate (p-NPA), 2-Thiobarbituric acid (TBA) and *N*-acetyl l-cysteine (NAC) were also obtained from Sigma Chemical Co.

### Preparation of protein stock solutions and determination of concentrations

All protein solutions were dialyzed at 4 °C using 0.1 M KCl solution and then filtered using Millipore syringe filter (0.22 μm). Concentration of protein samples were calculated using *ɛ*, molar extinction coefficient values of CA as 57,000 M^−1^ cm^−1^ at 280 nm, 43,824 M^−1^ cm^−1^ for BSA (at 280 nm) and 77,600 M^−1^ cm^−1^ for TTR (at 280 nm). All experimental protein samples were prepared using degassed 0.05 M potassium phosphate buffer (at pH 7.4) containing 0.1 M KCl.

### Protein covalent modification by MGO, GO and fructose

For the covalent modification of proteins with the AGEs, all protein samples were incubated in presence of MGO, GO and Fructose (1 mM each) in 0.05 M potassium phosphate buffer, pH 7.4 at 37 °C. For investigating the effect of AGEs, the protein samples were pre-exposed to NAC/SAC (0.25–1 mM) and then added MGO/GO/Fructose to it. The modified protein samples in such a way were further used for subsequent analyses.

### Estimation of carbonyl content using DNPH

Total protein carbonyl content was determined following the method described by Levine et al.^31^. Briefly, 2,4-dinitrophenyl hydrazine (DNPH) (0.1% w/v in 2N HCL) was added to MGO-treated protein samples and kept in dark for 1 h at room temperature to allow the formation of colored hydrazone product. Equal volume of 20% trichloroacetic acid (TCA) was then added to stop the reaction and centrifuged (5000 rpm for 15 min) to obtain a pellet. Unbound DNPH was removed by washing the pellets 2–3 times using 1 ml of ethyl acetate:ethanol (1:1 v/v) solution. Pellets were air-dried and dissolved in 6.0 M GdmCl (pH 7.0). Solubilised hydrazones were lastly measured at 370 nm and concentration of DNPH-derivatized proteins was determined using molar extinction coefficient of 22,000 M^−1^ cm^−1^.

### Measurement of AGE adduct fluorescence

This was carried out by measuring the AGE-specific fluorescence of the modified samples in a Perkin Elmer LS 55 Spectrofluorimeter in a 3 mm quartz cell with excitation and emission slits set at 10 nm^7^. For this, samples were excited at 370 nm and emission spectra were collected from 390 to 550 nm. Necessary blanks were subtracted for all samples.

### Measurement of di-tyrosine formation

Di-tyrosines have also been analyzed by measuring their specific fluorescence pattern in a Perkin Elmer LS 55 Spectrofluorimeter in a 3 mm quartz cell with excitation and emission slits set at 10 nm^32^. To check the formation of di-tyrosine moieties, protein samples were excited at 330 nm and emission spectra were collected from 350 to 500 nm. Necessary blanks were subtracted.

### Measurement of fluorescence spectra of proteins

Fluorescence spectra were measured in a Perkin Elmer LS 55 Spectrofluorimeter in a 3 mm quartz cell with excitation and emission slits set at 10 nm. Concentration of proteins used was 0.5 mg/ml (i.e. 15 μM for CA, 7.5 μM for BSA and 36 μM for TTR). Protein samples (CA, BSA and TTR) were excited at 295 nm, while the emission spectra were recorded from 300 to 500 nm.

### Measurement of ANS fluorescence spectra

For ANS binding assay the protein samples were excited at 360 nm and emission spectra were recorded from 400 to 600 nm. Concentration of proteins used was 0.5 mg/ml (i.e. 15 μM for CA, 7.5 μM for BSA and 36 μM for TTR). ANS concentration was kept 16-fold than that of protein concentration. Necessary blanks were subtracted for each sample. Each spectrum was repeated at least three times.

### Activity measurement of CA

Enzyme activity of CA was carried out by monitoring the hydrolysis of p-nitrophenyl acetate (pNPA) at 400 nm for 3600 s in 0.05 M phosphate buffer at 37 °C. The enzyme concentration used was 1 μM and substrate was kept 1 mM^33^.

### Circular dichroism (CD) measurements

Adducted protein samples were subjected to CD measurements using Jasco J-810 spectropolarimeter with at least three accumulations and proper blanks were subtracted from each of the readings. For Far-UV spectra, wavelength scans were recorded from 200 to 240 nm. Similarly for near-UV spectra, wavelength scans were recorded from 250 to 320 nm. Protein concentration used for CD measurement was 9 μM for CA, 4.5 μM for BSA and 21.5 μM for TTR. Cells of 0.1 and 1.0 cm path lengths were used for the measurements of far- and near-UV CD spectra, respectively.

### Dynamic light scattering (DLS) measurements

DLS measurements were carried out in a Malvern Zetasizer MicroV to obtain hydrodynamic diameter of unmodified and modified proteins at 37 ± 0.1 °C. Protein samples were filtered through a 0.22 µm filter. Measurements were made at fixed angle of 90° using an incident laser beam of 689 nm. Fifteen measurements were made for each sample with an acquisition time of 30 s at a sensitivity of 10%. Data were analyzed using Zetasizer software to get hydrodynamic diameter which is a measure of standard deviation of size of the particle. Protein concentration used was 30 μM.

### TEM imaging

Protein samples were placed on copper grids and air dried. Negative staining was done with 1.0% uranyl acetate and samples were again allowed to air dry. Finally, samples were examined under FEI Tecnai G2-200 kV HRTA transmission electron microscopy operating at 200 kV.

### Absorption spectroscopy studies

Absorption spectra of MGO and SAC co-incubated samples were measured using JASCO V-660 UV–Vis Spectrometer equipped with peltier controller in the wavelength range of 240–400 nm at 37 °C. Cell of 1 cm path-length was used for the measurement.

### 2-Thiobarbituric acid (2-TBA) method

2-TBA reagent (0.1 mM) was added to the samples and incubated at 90 °C for 1 h (adapted from Alam et. al., 2016 with minor modification)^34^. Thereafter, absorbance of the pink-colored complex was measured at 532 nm. Necessary blanks were subtracted during all the measurements.

### Cell lines and maintenance of cell culture

HT22 cells were grown in cell culture flasks containing DMEM growth media supplemented with antibiotic/antimycotic solution obtained from Himedia (catalog number-A002), 10% fetal bovine serum (Brazilian origin) and 0.25% Trypsin–EDTA (catalog number-25200-072) obtained from Gibco. The cells were maintained in a CO_2_ incubator (New Brunswick, Galaxy 170R, Eppendorf) at 37 °C supplied with 5% CO_2_ and relative humidity of about 98%.

### Cell viability assay

Onto a 96-well plate, HT22 cells were first seeded at a density of 5 × 10^3^ cells/well and incubated for 24 h. Seeded cells were treated with 15 μM of BSA, BSA and MGO (1 mM) mixture, and BSA, MGO and SAC/NAC (0.25 mM and 1 mM) for 24 h and 20 µl of MTT reagent (5 mg/ml stock) was then added to each of the well and kept for 4 h at 37 °C. Thereafter media was discarded from each well and 50 μl of DMSO was added to solubilize the formazan crystals. After incubation for 20 min in dark, absorbance was recorded at 570 nm using an microplate reader (Fluostar Optima, BMG Labtech, Ortenberg, Germany). Percent of cell viability was calculated using the equation:

$$Percent\,of\,cell\,viability = \frac{Mean\,absorbance\,of\,treated\,cells}{{Mean\,absorbance\,of\,vehicle\,treated\,cells}} \times 100.$$

### ROS analysis

HT22 cells (0.1 × 10^6^ cell/ml), under standard conditions, were seeded in a 12-well plate containing DMEM media and allowed to attach for 24 h. The attached cells were treated with 15 μM of BSA, BSA-MGO mixture, and BSA-MGO-SAC/NAC (0.25 mM and 1 mM) for 24 h and the media was removed. Following this, DCFH-DA dye (25 µM) was added to each well and incubated in dark for 30 min at room temperature. DCFH-DA deacetylates to DCFH, which further is converted into fluorescent compound DCF in presence of intracellular ROS. Unbound dye was removed by washing the cells with 1× PBS. Intracellular ROS generation was analysed using fluorescence microscopy by exciting the samples at 488 nm (blue laser irradiation). Fluorescence intensity of each image was further quantified using Image J software and plotted in a bar plot.

### Apoptosis measurements

The stages of apoptosis were assessed using Acridine orange (AO: 100 μg/ml)/Ethidium bromide (EB: 100 μg/ml) dye mixture following the method described by Takahashi et al. (2004). HT22 cells (0.05 × 10^6^ cells/well) were seeded in a 24-well plate containing DMEM media for 24 h for attachment. The attached cells were then treated with 15 μM of BSA, BSA and MGO (1 mM) mixture, and BSA, MGO and SAC/NAC (0.25 mM and 1 mM) mixtures for 24 h. Thereafter, the cells were then fixed with 4% formaldehyde and stained with 500 μl of AO/EB dye mixture (in 1× PBS). Cells were then kept in dark for 15 min and stain was washed with 1× PBS before visualizing under a fluorescence microscope (Evos, Floid). Fluorescence intensity of each image was further quantified using Image J software and plotted in a bar plot.

### Statistical analysis

All graphs were plotted using GraphPad Prism 5.04 software (GraphPad software, San Diego, USA). One-way ANOVA was used to compare the datasets. All analyses were carried out at 95% confidence level (CI) and considered to be significant at statistical probability (p-value) < 0.05.

### Ethics approval and consent to participate

Not applicable as no plant material or animal study is included in the study.

## Results

### SAC inhibits glycation of CA by MGO

**Figure 1:**
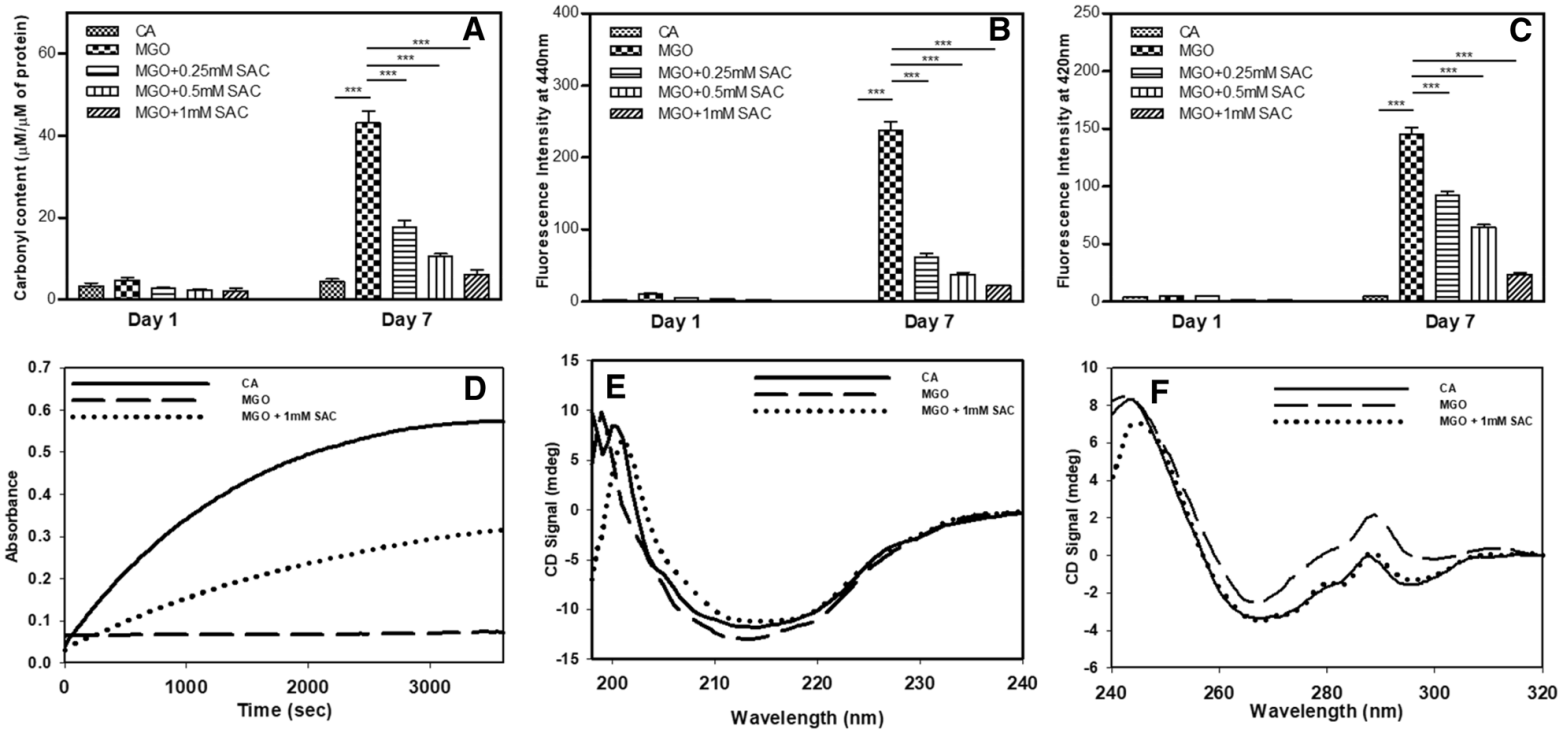
SAC suppresses glycation of CA by MGO. Effect of SAC on the total carbonyl content (**A**), MGO-adduct fluorescence (**B**) and di-tyrosine fluorescence (**C**). Activity status of MGO-modified CA in the absence and presence of SAC (**D**). Panels (**E**) and (**F**) represents the far-UV and near-UV CD of the modified CA in presence of SAC. The concentration of MGO used was 1 mM.

Since MGO is a dicarbonyl compound, it reacts with side-chain amino group of lysine to form an amide linkage leaving the other carbonyl group free. Therefore, the extent of adduct formation is directly proportional to the amount of free carbonyl group generated that can be detected using DNPH dye^35^. It is seen in the Fig. 1A that there is no significant increase in the total carbonyl content at day 1 of MGO-adducted CA while there is a sharp increase at day 7. However, upon addition of SAC, the carbonyl content decreases in a concentration-dependent manner (0.25–1 mM) indicating that SAC prevents glycation of CA. We have chosen day 7 for the analysis because there is no significant increase in the total carbonyl content of MGO-adducted CA till day 4 beyond which it gradually increases till day 7 that ultimately drops at day 8 (Supplementary Fig. S1). It has previously been reported that glycated peptides with MGO exhibit peculiar fluorescence emission at 440 nm when excited at 370 nm^7^. Furthermore, AGEs could also bring about changes in the overall conformation of protein resulting in dimer formation between two nearby tyrosine residues that emits fluorescence at 420 nm^32^. Following similar protocols, we have further measured the AGE-adduct specific fluorescence and di-tyrosine fluorescence of the modified protein samples in presence of SAC (Fig. 1B,C). Figure 1B shows that modified protein exhibits AGE-adduct specific fluorescence and the fluorescence intensity is reduced upon addition of different SAC concentrations. It is also seen in Fig. 1C that there is subsequent reduction in di-tyrosine fluorescence in presence of different concentrations of SAC.

Covalent adduct formation with MGO has been known to bring about conformational alterations that might render impairment of enzyme activity^7^. Protection of the CA by SAC from covalent adduct formation by MGO should therefore, be reflected at structural and functional levels. Enzymatic assay shows that there is 100% loss in functional activity of the MGO-adducted CA but is reversed by around 50% upon addition of SAC (1 mM) (Fig. 1D). Furthermore, it is also seen in Supplementary Fig. S2 that upon addition of different concentrations of SAC there is dose-dependent recovery of the enzyme activity (Supplementary Fig. S2). Far- and Near-UV CD measurements revealed different structural alteration both in the secondary and tertiary levels upon modification by MGO (Fig. 1E,F). There is no significant change in the CD signal at around 218 nm (Fig. 1E) which is a signature of β-sheeted proteins. Figure 1F also shows that there is an increase in signals at 275 nm and 290 nm. Bands at 275 nm and 290 nm reflect asymmetry of Tyr and Trp residues, respectively. This increase in the intensities at 275 nm and 290 nm might be because of changes in the relative orientation of the planar chromophores in these aromatic residues, indicating an overall alteration in the micro-environment of the chromophoric groups. This is further supported by the observed hyperchromicity of the Trp fluorescence spectra upon modification indicating that the micro-environment of the Trp is perturbed by glycation (Supplementary Fig. S3A). It is also seen in this figure that addition of SAC reverses the structural alteration caused by MGO. We have further probed the packing of the hydrophobic core of modified protein by performing ANS binding assay because ANS is a hydrophobic dye that can specifically bind to the hydrophobic clusters of the protein when exposed to the solvent. ANS binding was evident from the hyperchromicity accompanied with a slight blue shift in MGO-modified CA in absence of SAC (Supplementary Fig. S3B). Interestingly, upon addition of SAC, the native state protein integrity remains intact as there is nearly identical ANS binding behavior between native CA and SAC treated samples.

### SAC prevents formation of cross-linked oligomers in CA

**Figure 2:**
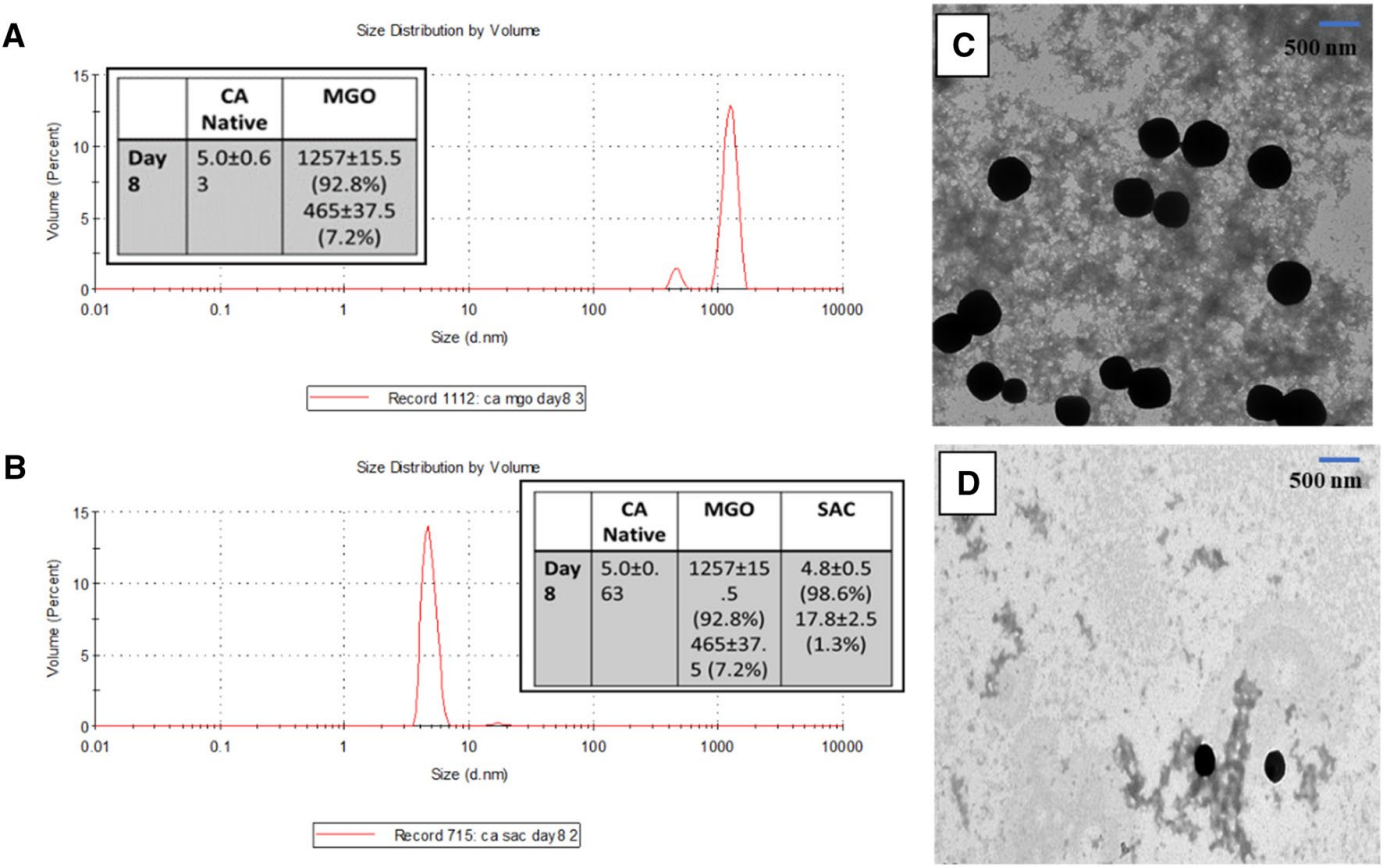
Effect of SAC on the nature of oligomers formed by MGO-modification of CA. Size distribution by volume of MGO-modified CA in absence (**A**) and presence (**B**) of SAC. TEM images of MGO modified CA in absence (**C**) and presence (**D**) of SAC.

Since, protein glycation has been shown to result in protein crosslinks in many proteins (although not true in general)^36,37^, we were curious to investigate what effect SAC and MGO have in oligomerization status of CA. Using DLS, we have systematically analyzed the oligomer formation of the MGO-modified protein in absence and presence of SAC. Table 1 depicts the size distribution by volume recorded at different time frames. Raw data of the DLS measurements are also given in Supplementary Fig. S4. It is seen in Table 1 that formation of multimers/oligomers appears at day 8. Since, formation of oligomers initiates from day 8, we analysed oligomeric properties in presence of SAC at day 8 only. Figure 2A,B shows that addition of SAC results in the prevention of oligomer formation. For instance, in presence of 1 mM MGO, there was formation of multiple oligomers ranging from 400 to 1200 nm in size. However, in the presence of 1 mM SAC, there was appearance of the native CA with a volume fraction of 98% while only 0.5–2% are left as oligomeric species. TEM images also implicates that there is amorphous aggregates of the MGO-modified proteins which upon pre-exposure to SAC, results in disappearance of the oligomeric species (Fig. 2C,D). The overall results indicate that SAC prevents CA against formation of cross-linked oligomers.

**Table 1:** Hydrodynamic diameter of MGO-modified CA at different time frames. The vales in parentheses represents percent volume fractions of the species present.

	CA native	MGO treated
Day 1	4.845 ± 0.41	4.731 ± 0.34
Day 2	4.890 ± 0.33	4.899 ± 0.63
Day 3	4.740 ± 0.70	4.790 ± 0.85
Day 4	4.873 ± 0.40	4.758 ± 1.19
Day 5	4.838 ± 0.52	4.921 ± 0.57
Day 6	4.855 ± 0.60	4.420 ± 0.76
Day 7	4.667 ± 0.36	5.219 ± 1.11
Day 8	5.008 ± 0.63	1257 ± 15.5 (92.8%) 465.4 ± 37.5 (7.2%)

### SAC fails to reverse modification of MGO-adducted CA

**Figure 3:**
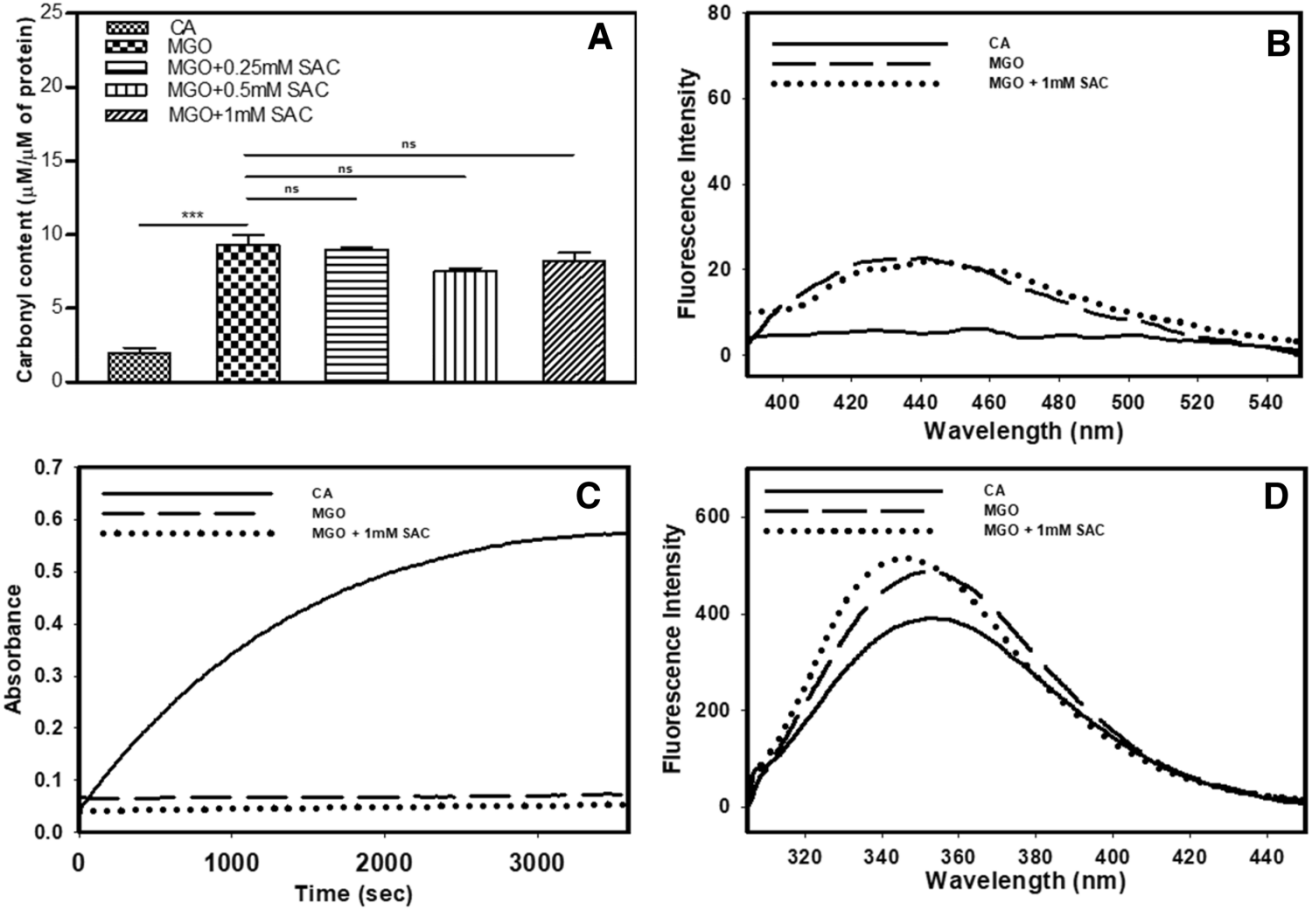
Effect of SAC on the reversibility of covalently modified CA by MGO. Total carbonyl content (**A**) and AGE-specific fluorescence spectra (**B**) of CA pre-exposed to MGO in presence of SAC. Panels (**C**) and (**D**) represents enzyme activity status and tryptophan fluorescence spectra (**D**) respectively of the modified samples in presence of SAC.

Next, we have investigated the effect of SAC on the reversal of pre-adducted CA. Figure 3A,B show that the total carbonyl content and AGE-adduct fluorescence intensity of the MGO-adducted CA in the absence and presence of SAC are almost identical indicating that SAC could not influence the pre-adducted CA. Enzymatic activity analysis further confirmed that SAC could not reverse the functional activity of MGO-modified CA as the percent activity in absence and presence of SAC are nearly similar (Fig. 3C). Tryptophan fluorescence spectra of the modified CA with and without SAC are also not significantly different indicating the failure of SAC to restore structural perturbations induced by MGO (Fig. 3D).

### Mechanism of inhibition of covalent modification of CA by MGO

**Figure 4:**
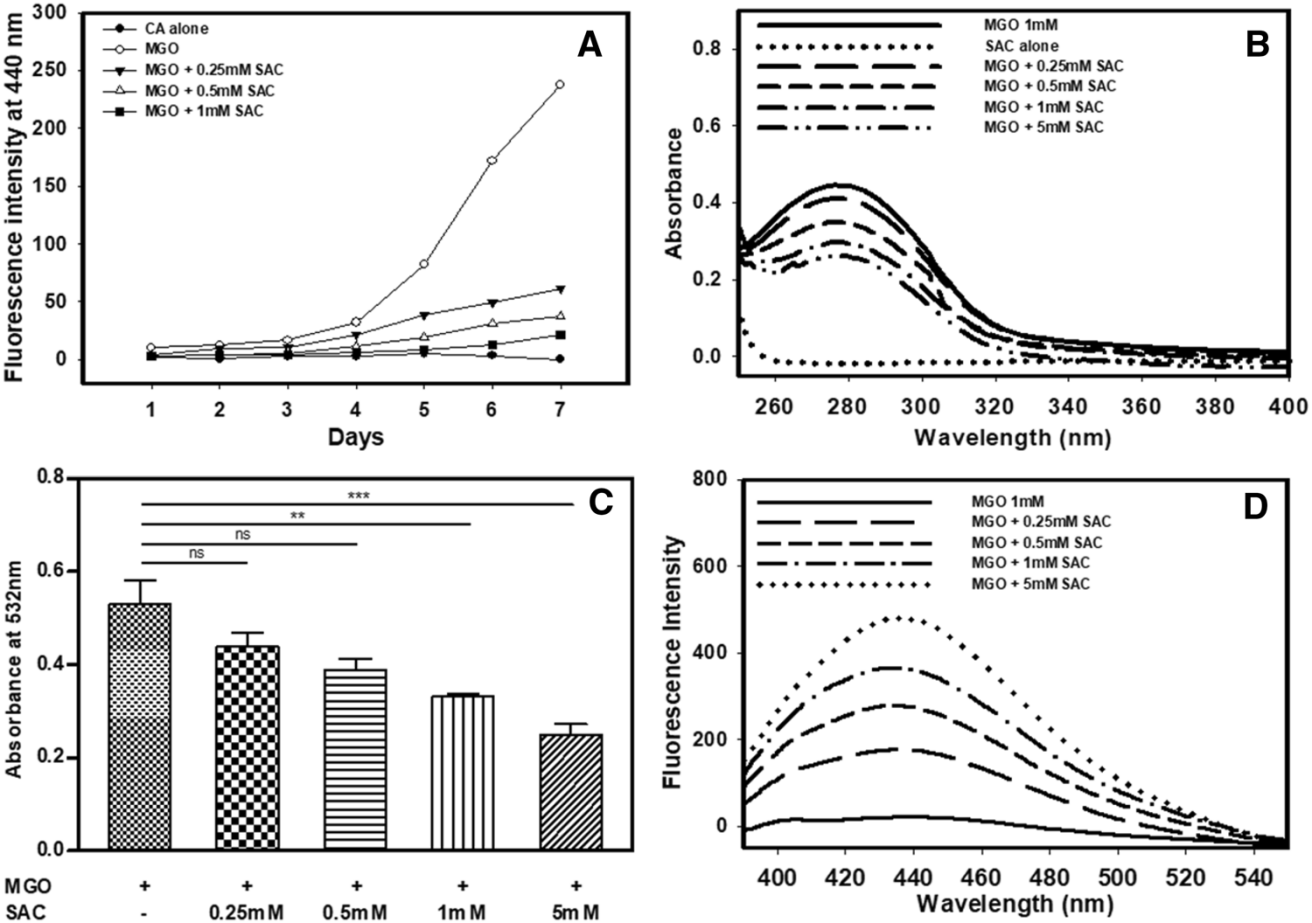
Mechanism of SAC-induced inhibition of CA glycation by MGO. Time dependent reactivity of AGE specific fluorescence of the modified CA in presence of different concentrations of SAC (**A**), absorbance spectra of MGO upon incubation with different concentrations of SAC (**B**), decrease in TBA-MGO product with increasing concentration of SAC (**C**) and AGE specific fluorescence of different co-incubated MGO-SAC mixtures (**D**).

First of all, we measured the time dependent changes in the AGE-adduct fluorescence in the presence of different SAC concentrations. Analysis of this time dependent AGE-adduct fluorescence (Fig. 4A) of the modified CA in presence of SAC revealed that SAC treatment decreases the reactivity of MGO toward CA. The decrease in reactivity might be due to the formation of complex between MGO and SAC. To investigate for this possibility, we have firstly co-incubated MGO and SAC and measured the product formation using two different methods, one by directly measuring the MGO concentration and the other using TBA method (Fig. 4B) (see “Discussion” for detail)^38,39^. We observed that there was decrease in the absorbance of MGO upon addition of different concentration of SAC (Fig. 4C). It is also seen in Fig. 4C that there is an increase in TBA-MGO product in absence of SAC but is decreased upon addition of SAC in a concentration dependent manner. Figure 4D also shows that the MGO-SAC complex exhibits AGE-adduct specific fluorescence similar to that of the glycated proteins. If complex between MGO and SAC is indeed formed, there should be a good correlation between the fall in MGO concentration with the rise in complex formation. For this we have analysed the plot of absorbance at 277 nm versus fluorescence intensity at 440 nm (see Supplementary Fig. S5). We observed a positive linear association with R^2^ value of 0.95. Taken together the results indicate that SAC has the potential to trap MGO.

### Inhibitory effect of SAC is true to other glycating agents

**Figure 5:**
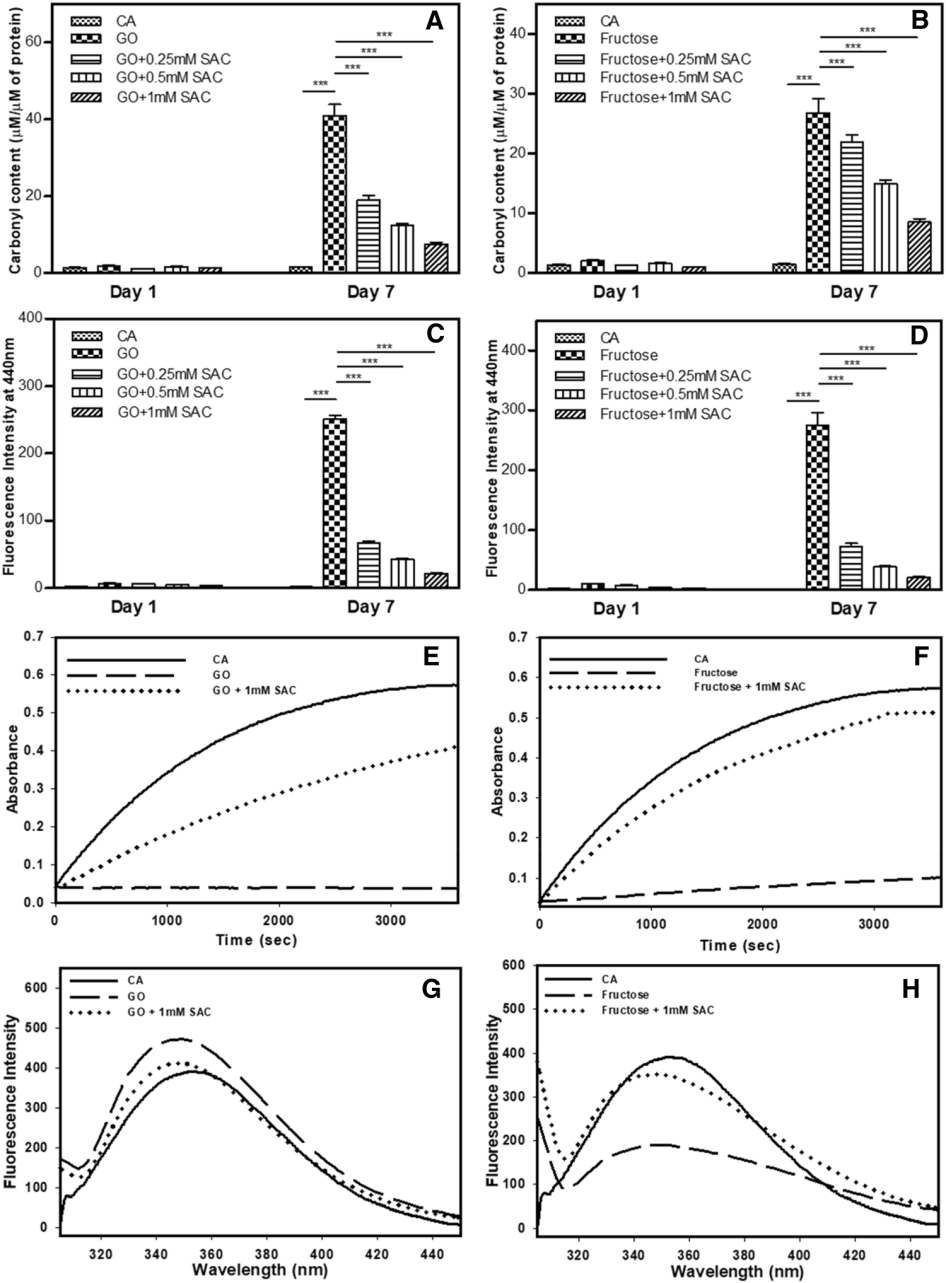
Effect of SAC on the glycation of CA induced by GO and fructose. Total carbonyl content (**A**,**B**), AGE-specific fluorescence (**C**,**D**), activity status (**E**,**F**) and tryptophan fluorescence spectra (**G**,**H**) of CA glycated by GO and fructose in presence of SAC. The concentration of GO and fructose used were 1 mM each.

To get a better insight if SAC is effective against carbonyl stress in general, we extended our work on two more glycating agents, glyoxal (GO) and fructose. We have included fructose to investigate if SAC could also inhibit glycation induced by AGE precursor, reducing sugars. First of all, we carried out modification of CA by GO (1 mM) and fructose (1 mM) and the modified proteins have been analyzed for the extent of glycation by measuring total carbonyl content (Fig. 5A,B) and AGE-adduct fluorescence (Fig. 5C,D). It is seen in the figures that there is an increase in the total carbonyl content as well as AGE-specific fluorescence in the presence of both GO or fructose indicating that the protein is glycated. However, upon addition of SAC, there is 70–90% decrease in the AGE-adduct fluorescence intensity with varying concentration of SAC. The results indicate that SAC inhibits glycation of CA by both GO and fructose. To further confirm, we have also pre-exposed CA to different concentrations of SAC and glycating agents are then added. Such co-incubated samples have been analysed for tryptophan fluorescence to assess the structural alteration (Fig. 5G,H) and enzyme activity (Fig. 5E,F). It is seen in Fig. 5E,F that there is 70% and 90% reversal in the enzyme activities upon addition of SAC in case of GO and fructose respectively. Nearly identical Trp fluorescence spectra as compared to the unmodified control (Fig. 5G,H) further support the premise that SAC inhibits structural alteration caused by both the GO and fructose.

### Inhibitory effect of SAC in not confined to CA

**Figure 6:**
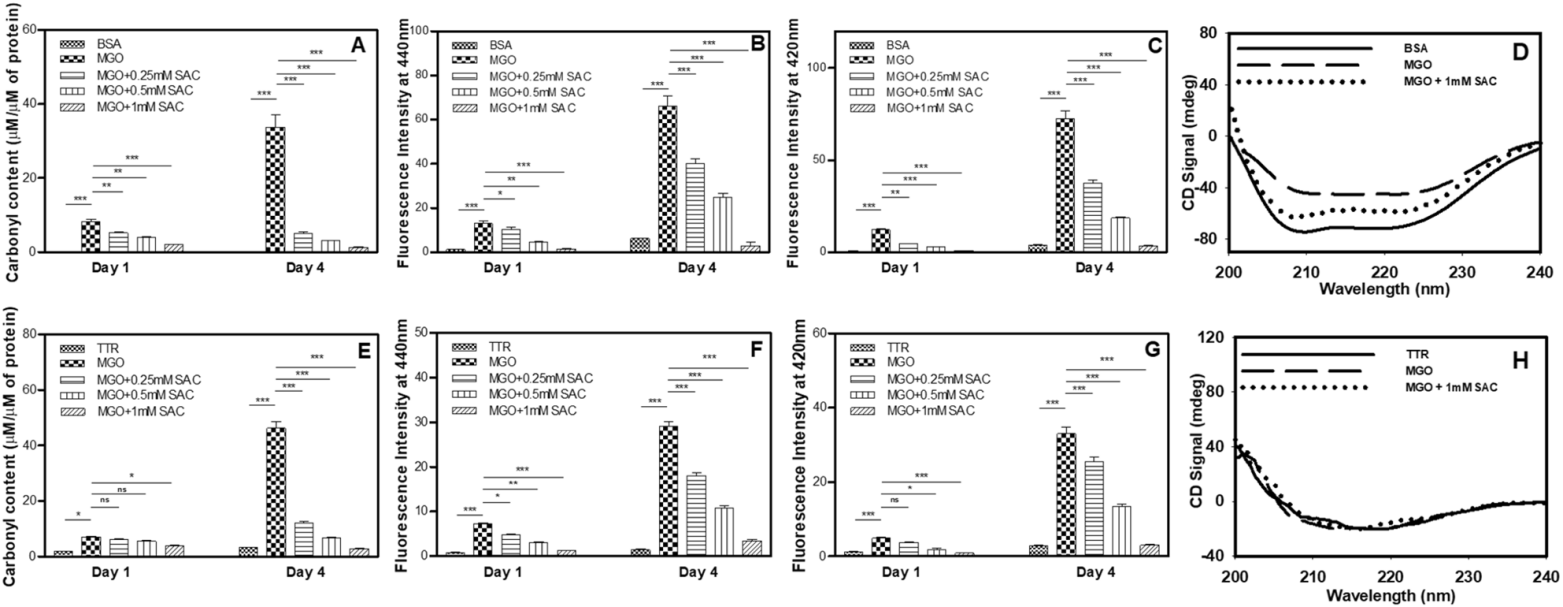
Effect of SAC in preventing the MGO-induced glycation of BSA and TTR. Total carbonyl content (**A**) AGE specific fluorescence (**B**) and di-tyrosine fluorescence spectra (**C**), far-UV CD spectra (**D**) of MGO-modified BSA. Total carbonyl content (**E**) AGE specific fluorescence (**F**) and di-tyrosine fluorescence spectra (**G**), far-UV CD spectra (**H**) of MGO-modified TTR.

To investigate the generality of the protective effect of SAC against MGO-induced covalent modification, we extended our work on two more serum proteins, BSA and TTR. For this, both the proteins have been exposed to 1 mM MGO in the absence and presence of SAC. Figure 6A,E show that SAC inhibits glycation of both BSA and TTR as evident from the decrease in the total carbonyl content. We again confirmed these results by measuring other parameters associated with glycation including AGE-adduct fluorescence (Fig. 6B,F) and di-tyrosine fluorescence (Fig. 6C,G). Far-UV CD measurements of BSA (Fig. 6D) shows that SAC treatment brings the altered spectral behavior of MGO-modified BSA back to its native state. However, in case of TTR, there is nearly identical spectra of modified protein as compared to that with native TTR (Fig. 6H). Taken together, the results led us to believe that the ability of SAC to inhibit MGO-induced covalent adduct formation in proteins is true in general.

### SAC suppresses cytotoxic effect of MGO-adducted BSA in HT-22 cells

**Figure 7:**
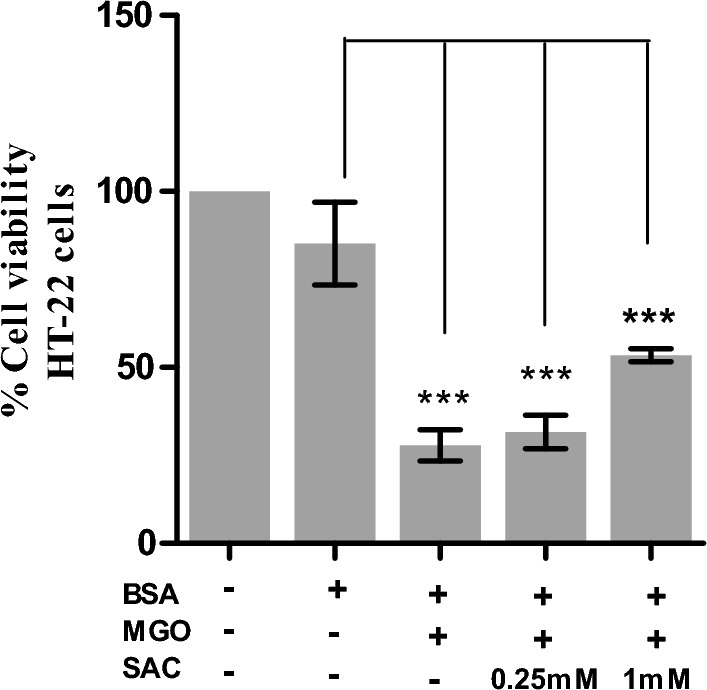
Effect of SAC on the cytotoxicity of HT22 cells induced by glycated BSA. Percent viability of cells in the presence of MGO-BSA, and MGO-BSA-SAC mixtures. The concentrations of BSA and MGO used were 15 μM and 1 mM respectively.

For this, we examined the cytotoxic effect of MGO by using glycated BSA. Briefly, cells were exposed to BSA alone (control), MGO-BSA, MGO-BSA-SAC (different concentrations of SAC) mixtures. Cells were grown for 24 h and viability of the cells were analysed using MTT assay (Fig. 7). It is seen in figure that addition of BSA does not largely affect the percent cell viability while treatment with MGO-BSA results in drastic decrease in the percent cell viability (around 70%). It is also seen in this figure that in the presence of MGO-BSA-SAC (with 1 mM SAC), there was around 20% increase in the cell viability. However, there was no significant effect in the presence of MGO-BSA-SAC (with 250 μM SAC).

### SAC reduces ROS generation in HT22 cells induced by MGO-adducted BSA

**Figure 8:**
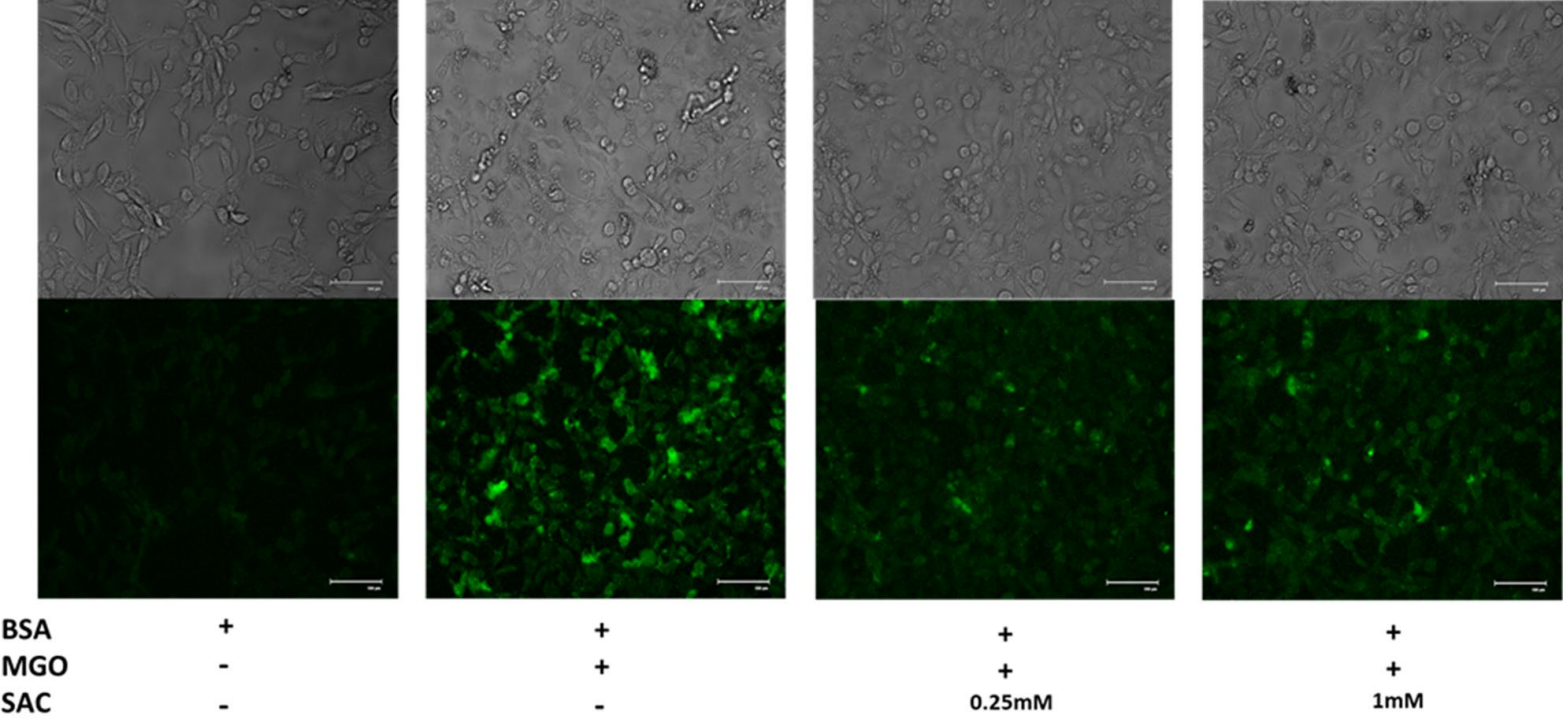
Effect of SAC on ROS levels induced by glycated BSA. Fluorescence microscopy of ROS images in HT22 cells in the presence of MGO-BSA, and MGO-BSA-SAC mixtures. The concentrations of BSA and MGO used were 15 μM and 1 mM respectively.

We have further analyzed the BSA, MGO-BSA and MGO-BSA-SAC treated cells for ROS levels using DCHF-DA dye. DCHF-DA is a fluorescent dye and is cleaved by intracellular esterases at the two ester bonds, producing H2DCF. This non-fluorescent product accumulates in cells and subsequent oxidation yields a fluorescent product, DCF. Fluorescence microscope images show large increase in ROS levels with MGO-BSA treatment but is reduced upon addition of SAC (Fig. 8). Bright field images also show that the cell morphologies are not largely perturbed by the presence of MGO-BSA or MGO-BSA-SAC (Fig. 8). Relative ROS levels have also been quantified by analysing the fluorescence intensity of the images (see Supplementary Fig. S6). It is seen in the figure that there is 40–75% decrease in the ROS levels upon addition of SAC compared to the cells treated with BSA-MGO.

### SAC prevents apoptosis of HT-22 cells induced by MGO-adducted BSA

**Figure 9:**
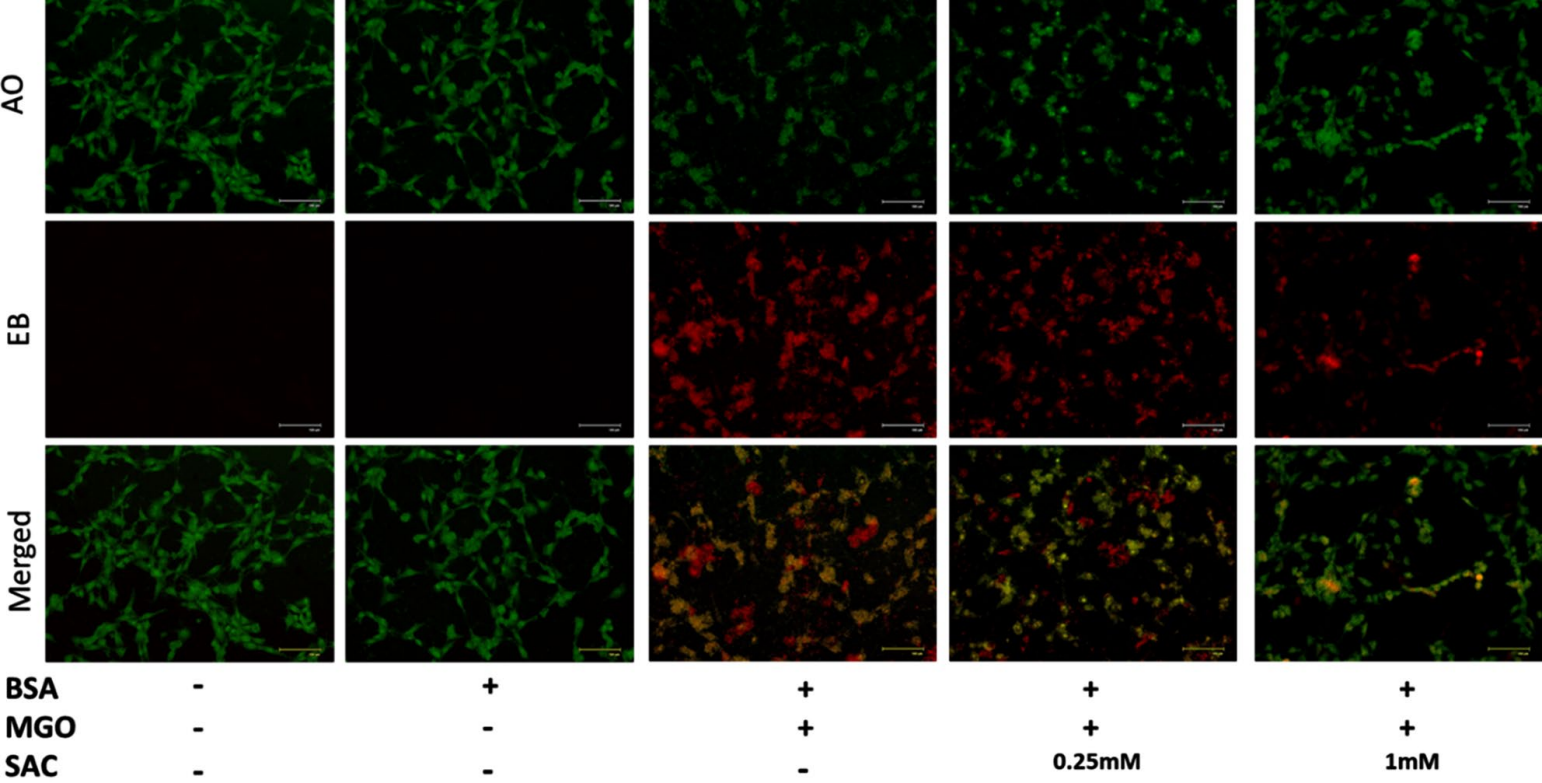
Effect of SAC on the apoptotic status of HT22 cells induced by glycated BSA. Fluorescence microscopy image of HT22 cells representing apoptotic status in the presence of MGO-BSA, and MGO-BSA-SAC mixtures. Cells stained with acridine orange, ethidium bromide, and AO/EB mixture are labeled as AO, EB and merged respectively. The concentrations of BSA and MGO used were 15 μM and 1 mM respectively.

We further analyzed the MGO-BSA treated cells for the induction of apoptosis with the help of AO/EB dye (Fig. 9). For this, cells were again treated with MGO-BSA or MGO-BSA-SAC and allowed to grow for 24 h and then stained with AO/EB dye. AO penetrates normal and early apoptotic cells with intact membranes, fluorescing green when bound to DNA. EB, on the other hand, only enters cells with damaged membrane (late apoptotic and dead cells), emitting orange-red fluorescence. As evident, the fluorescence microscopic images show slight/no sign of apoptosis in BSA alone treated cells (Fig. 9). Indeed, the cells displayed uniform bright green fluorescence with intact nuclear architecture, attributing to healthy viable cells. In case of MGO-BSA treatment, the number of late apoptotic cells increased as evident from disappearance of bright green fluorescence and appearance of bright orange-red fluorescence. However, in case of MGO-BSA-SAC treated cells, there was decrease in orange-red fluorescence and appearance of bright green fluorescence in a concentration-dependent fashion. Furthermore, relative reduction in percent apoptotic cells have also been quantified by analysing the green/red fluorescence intensity of the images (see Supplementary Fig. S7). It is seen in the figure that there is ~ 10–40% decrease in the number of apoptotic cells upon addition of SAC compared to the cells treated with BSA-MGO.

### NAC inhibits glycation of proteins by MGO

**Figure 10:**
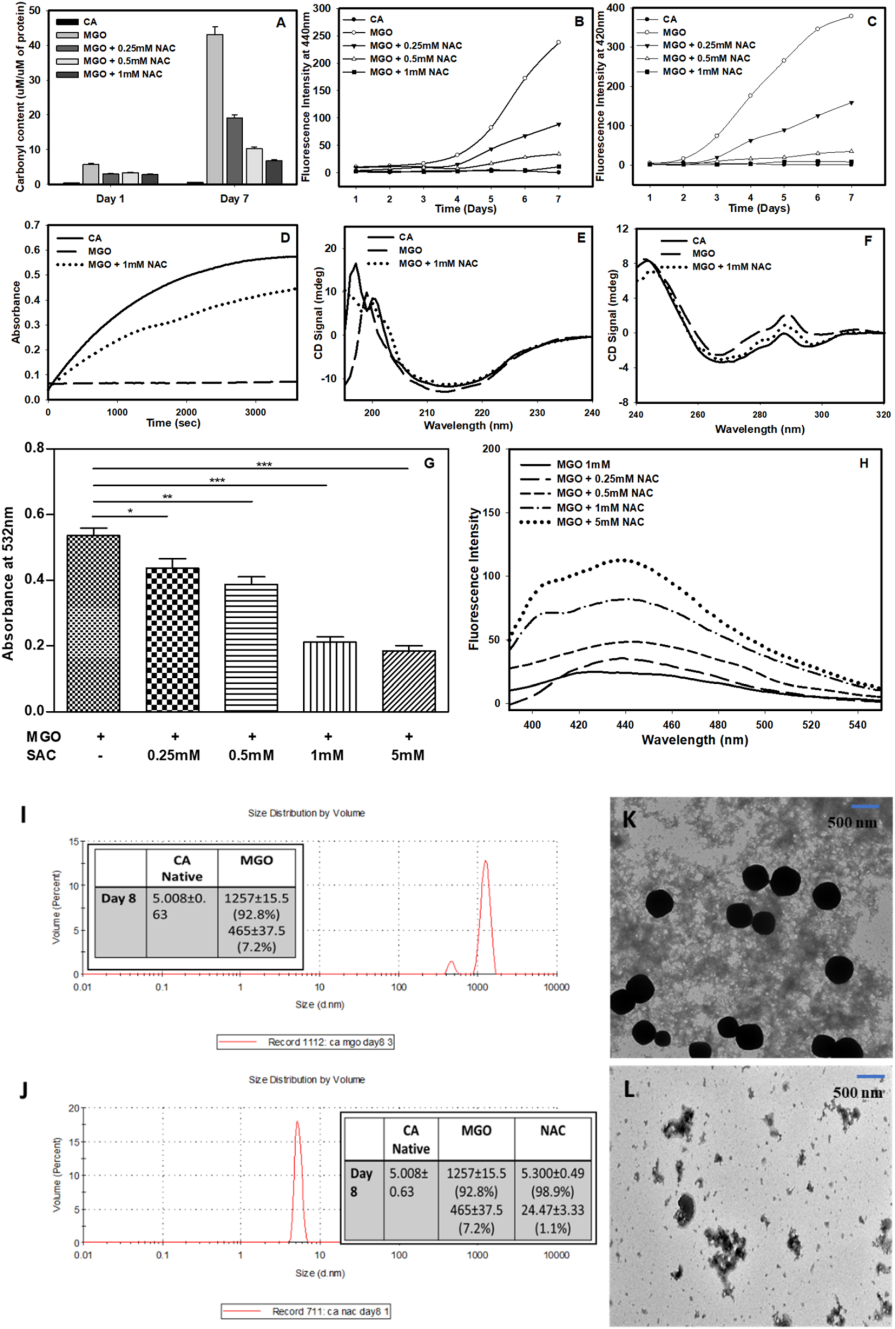
Effect of NAC in suppressing the glycation of CA by MGO. Effect of NAC on the total carbonyl content (**A**), time dependent MGO-adduct fluorescence (**B**) and time dependent di-tyrosine fluorescence (**C**), activity status (**D**), far-UV (**E**) and near-UV CD (**F**) spectra of MGO-modified CA in the absence and presence of NAC. Decrease in MGO-TBA product in presence of NAC (**G**), increase in AGE specific fluorescence (**H**) in presence of different concentrations of NAC. Size distribution by volume of MGO-modified CA in absence (**I**) and presence (**J**) of NAC. TEM images of MGO modified CA in absence (**K**) and presence (**L**) of NAC.

To investigate the effect of NAC against MGO-induced covalent modification of proteins, we first assessed the change in total carbonyl content, AGE-adduct fluorescence and di-tyrosine fluorescence. Figure 10A shows gradual decrease in the total carbonyl content of MGO-treated CA in presence of NAC in a concentration-dependent fashion. Concomitantly, there is decrease in both AGE-adduct fluorescence (Fig. 10B) and di-tyrosine fluorescence (Fig. 10C) in a NAC concentration dependent manner indicating that NAC inhibits MGO-induced protein glycation. Loss of activity with MGO treatment and eventual recovery (around 75%) upon addition of NAC also support the premise that NAC inhibits glycation of CA by MGO (Fig. 10D). Furthermore, it is also seen in figure S8 that upon addition of different concentrations of NAC there is dose-dependent recovery of the enzyme activity (Supplementary Fig. S8). Far-UV and near-UV CD spectra of the MGO-modified CA in the presence of NAC have also been procured to assess the structural alterations (Fig. 10E,F). It is seen in figure that there is reduction in the secondary (as evident from the decrease in CD signal at 218 nm) (Fig. 10E) and tertiary contacts (as evident from the decrease in CD signal at 275 and 290 nm regions) (Fig. 10F) with MGO treatment and reversal of the spectral property nearly identical to that of the native protein upon addition of NAC. The results indicate that similar to SAC, NAC could also prevent structural alterations brought about by the MGO-induced modification. It is also seen in Fig. 10G that there is a decrease in the MGO-TBA product upon addition of NAC in a concentration dependent manner. Figure 10H also shows that the MGO-NAC complex exhibits AGE-adduct specific fluorescence and there is observed hyperchromicity with increasing concentration of NAC. The results indicate that similar to SAC, NAC also prevents MGO-induced protein modification by virtue of its potential to form complex with MGO.

We again analysed if the oligomerization of CA because of MGO-induced modification is suppressed in presence of NAC by performing DLS measurements (Fig. 10I, J) and TEM imaging (Fig. 10K,L). Figure 10I, J shows that there is appearance of monomeric species with 5.3 nm in size (volume fraction of 99%). There is only 1% species existing in the aggregated state with 25 nm in size. Furthermore, TEM images of the modified protein samples also show that there is complete disappearance of the large size spherical species and also reduction in the amyloidogenic oligomeric species upon addition of NAC (Fig. 10K,L).

### NAC protects HT22 cells from the MGO-induced cytotoxic effects

**Figure 11:**
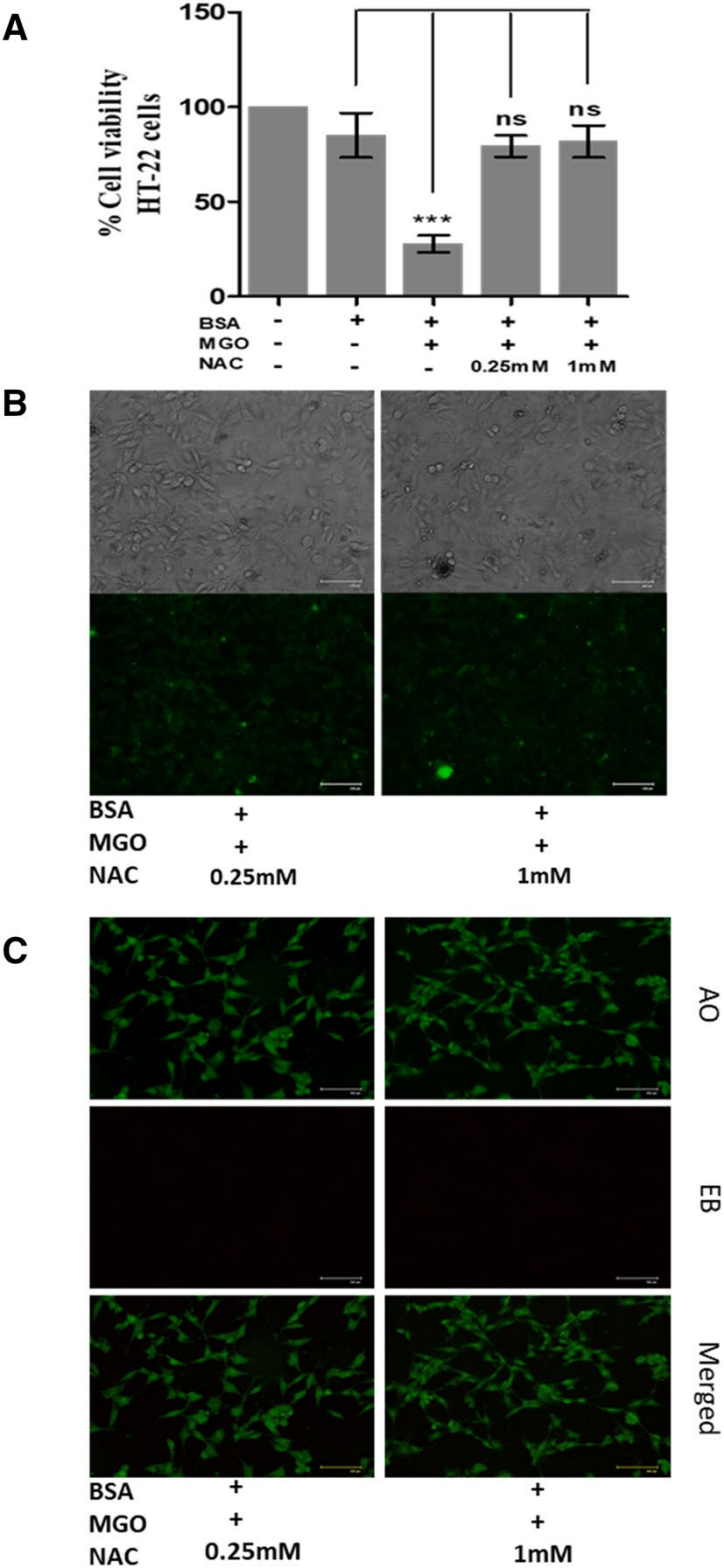
Effect of NAC against MGO-induced cytotoxicity in HT22 cells. Percent viability of cells in the presence of MGO-BSA, and MGO-BSA-NAC mixtures (**A**). Fluorescence microscopy of ROS images (**B**) and apoptotic status (**C**) of HT22 cells in the presence of MGO-BSA, and MGO-BSA-NAC mixtures.

Using similar experimental conditions as in case of SAC treatment, the effect of NAC on HT22 cells have also been examined. Firstly, we analyzed the suppressive ability of NAC against the cytotoxic effect of MGO. For this, cells were exposed to MGO-BSA-NAC (different concentrations of NAC) and measured cell viability. The results are then compared with that of the previously measured values in presence of MGO-BSA. It is seen in Fig. 11A that there is around 70% increase in cell viability as compared to MGO-BSA samples upon addition of NAC. Fluorescence microscope images further show large decrease in the ROS level as compared to the MGO-BSA control upon addition of NAC indicating a marked decrease in the ROS levels (Fig. 11B). Relative ROS levels have also been quantified by analysing the fluorescence intensity of the images (see Supplementary Fig. S9). It is seen in the figure that there is ~ 100% decrease in the ROS levels upon treatment with NAC as compared to the cells treated with BSA-MGO. Figure 11C also shows that there is complete reduction of orange-red fluorescence and appearance of more green fluorescence as compared to the MGO-BSA treated cells indicating that NAC is rescuing cells from apoptosis. Furthermore, upon quantifying the relative reduction in percent apoptotic cells it is seen that there is ~ 98–100% decrease in the number of apoptotic cells upon addition of NAC compared to the cells treated with BSA-MGO (Supplementary Fig. S10). The results confirmed that similar to SAC, NAC is also a potential anti-glycating agent.

## Discussion

Of all the organosulfurs present in garlic, SAC is the most abundant component^23^. Therefore, we have first of all investigated the anti-glycating potential of SAC on CA^28–30^. Our results in Fig. 1A–F and Supplementary Fig. S2A,B indicate that SAC protects CA from the covalent modification by MGO in terms of the extent of glycation, AGE-specific fluorescence, di-tyrosine fluorescence, structural and functional consequences. Since MGO is a dicarbonyl compound, adducted MGO has one free carbonyl that can attack lysine residues on other polypeptide chain forming cross-linked oligomers. Using DLS, we have firstly, monitored the time-frame for the appearance of such oligomers (Table 1 and Supplementary Fig. S4). Indeed, there was no increase in the hydrodynamic diameter of CA up to day 7 but on day 8, there was appearance of oligomeric species. Therefore, all other measurements and analyses of oligomers in presence of SAC have been carried at day 8 only. As expected, SAC prevents formation of oligomers as evident from the appearance of monomeric species in the SAC treated samples (Fig. 2A–D). In order to further investigate the behavior of the glycation kinetics in absence and presence of SAC, we further performed the measurement of time-dependent kinetics of glycated CA (Supplementary Fig. S11). We observed that in absence of SAC, the oligomerization of glycated protein exhibits a conventional sigmoidal nature of aggregation curve consisting of lag phase, log phase and the final aggregates. Interestingly, it appears that glycated CA completes the aggregation kinetics by 11th day. It is also evident in the figure that in presence of SAC there was complete absence of the logarithmic as well as final aggregates formed indicating the potent inhibitory effect of SAC against aggregation of glycated CA (Supplementary Fig. S11). In support previously, it has been shown that aged garlic extract and SAC could prevent crosslinking of BSA and lysozyme induced by glucose and MGO and subsequently reduces the formation of CML^40^. Our data on Figs. 5A–H, and 6A–H further revealed that the effect of SAC is independent of the protein or glycating used. Taken together, the results confirmed that SAC protects proteins against MGO-induced glycation.

Next, we investigated what effect SAC has on the already MGO-adducted CA. For this, we exposed CA with MGO so as to allow covalent modification to take place and then added SAC in the samples. We then analyzed the effect of SAC by examining 3 parameters, i.e., extent of glycation (Fig. 3A,B), enzyme activity (Fig. 3C) and structural consequences (Fig. 3D). It is evident from the figures that there is no significant effect of SAC on the total carbonyl content, AGE-specific fluorescence and consequently no reversal of enzyme activity or native state structure compared to the glycated controls. Thus, all measurements collectively revealed that SAC does not have any significant effect on the reversal of MGO-adducted CA (Fig. 3). Taken together, the results led us to believe that SAC exhibits differential behaviors on the adducted and non-adducted CA.

The differential effect of SAC, i.e., failure to reverse the MGO-bound CA (Fig. 3) but its suppressive potential when CA is pre-exposed to SAC (Figs. 1, 2) suggest that SAC might not be allowing binding of MGO to CA. A time-dependent decrease in the AGE-specific fluorescence in presence of SAC indicates that it perhaps works by lowering the reactivity of MGO to CA (Fig. 4A). Previous studies have demonstrated that there was reduction in the reactivity of MGO by forming complexes with certain amino acids, short peptides, phytochemicals, etc^41,42^. Therefore, we thought it would be worthwhile to investigate if SAC forms covalent adduct with MGO thereby not allowing the MGO to interact with the protein. A more direct way of analyzing complex formation is by measuring the amount of free MGO upon co-incubation with SAC. We monitored the MGO concentration by measuring its absorbance at 277 nm as MGO has an absorption maximum at this wavelength. It is evident in Fig. 4B that upon increasing SAC concentration, the absorbance of MGO (at 277 nm) decreases (Fig. 4C) indicating the formation of MGO-SAC complex. Another method for measuring the concentration of free MGO involves the use of 2-thiobarbituric acid (TBA) as a probe^38,39^. TBA reacts with MGO species generating a colored product with an absorbance maximum at 532 nm. It is speculated that MGO on binding with SAC would remain unreactive to TBA thereby resulting in the decrease in absorbance compared to the samples of MGO alone. As expected, MGO and SAC co-incubated samples show gradual decrease in absorbance at 532 nm with increasing concentration of SAC (Fig. 4B). Thus, our results led us to believe that SAC forms complex with MGO directly. In the second experiment, we excited the complex at the same wavelength conventionally used to detect glycated proteins and monitored the emission spectra (Fig. 4D). We observed identical emission spectral behavior as that of the glycated proteins and there was hyperchromicity with increasing concentration of SAC. The results further confirmed that the MGO-SAC complex thus formed is a conventional amide linkage type. We conclude that SAC inhibits MGO-induced covalent modification of CA by virtue of its potential to form amide linkage complex.

Cellular glucose uptake is highest in the neuronal cells^43,44^ and MGO or GO are the potential biomarkers of myocardial infarctions^45^. Furthermore, AGEs are not only synthesized in the neuronal cells but can cross blood brain barrier and accumulate in brain cells^46^. With these notions, we carried out cellular studies of the effect of SAC as antiglycation agent in HT22 cells (a mouse hippocampal neuronal cell line). Previously, glycated BSA has been used to study glycation effect in cellular and animal models. Similarly, in the present study, first of all, we glycated BSA with MGO and this glycated protein is used to induce cytotoxicity to the cells. It is seen in Fig. 7 that there is a decrease in the cell proliferation upon treatment with glycated BSA and is reversed upon addition of SAC. Accumulation of glycated proteins are known to cause oxidative stress^47,48^ and eventual apoptosis of cells^49,50^. For this we have further analyzed the effect of SAC on ROS level (Fig. 8) and extent of apoptosis (Fig. 9). It is seen in figure that glycated BSA brings about large increase in the ROS level and a concomitant increase in the extent of apoptosis. Indeed, treatment with BSA-MGO results in the early apoptotic to late apoptotic cells as evident from the appearance of yellow-green and bright red fluorescence respectively. However, upon addition of SAC, there is large reduction in ROS levels (Fig. 8) and concomitant recovery of healthy cells (Fig. 9). Thus, SAC protects HT22 cells against MGO-induced toxicities.

SAC is an organosulfur compound and therefore, we have additionally investigated the effect of NAC (another organosulfur present in garlic)^51^ as anti-glycating agent (Fig. 10). Our results in Fig. 10A–L indicate that NAC prevents CA from covalent modification by MGO and hence protects structure and functional integrity. Similar to SAC, the mechanism involves complex formation between MGO and NAC via amide linkage type of bonding. It is also evident in Fig. 11A that NAC has robust effect of protecting cell viability against the cytotoxicity caused by BSA-MGO mixture. Consequently, there is large protection against apoptosis (Fig. 11C) by virtue of its effect to lower oxidative stress (Fig. 11B) caused by the glycated protein. The results on NAC and SAC further conclude that organosulfurs are potent anti-glycating agents.

Diabetic patients require stringent control and proper management throughout their life, failure of which results in chronic pathological conditions including neurodegeneration and cardiovascular complications etc. The current regime for the management of diabetes focusses on regulating blood sugar levels^52^. Around 26% of the total diabetic patients are insulin users^53^. Therapy with human insulin analogue is found to be dramatically effective in lowering blood glucose in patients. Other drugs include Metformin, Sulfonylureas, Glinides, Dipeptidyl peptidase-4 (DPP-4) inhibitors^54,55^ and glucagon like peptide-1 (GLP-1) receptor agonists etc. Since, protein, DNA and lipid glycation is a major pathological event associated with diabetes, our results indicate that organosulfurs should be employed as anti-glycating agent. In particular, NAC is an FDA approved drug for the treatment of triple negative breast cancer^56^. It is also used to treat acetaminophen poisoning^57^. Interestingly, in addition to this present study, NAC has also been reported to reduce oxidative stress caused by glycated proteins in animal and cellular models^58,59^. Furthermore, studies also support that NAC is a promising drug for diabetes-related cardiovascular complications including, high blood pressure^60^, platelet monocyte conjugation^61^, cardiac function^62,63^, congenital heart defects^64^, etc. Therefore, attempt should be made to repurpose NAC for the treatment of diabetes as an adjuvant therapy or separately.

## Conclusions

Our study indicates that organosulfurs, NAC and SAC exhibit strong anti-glycating potential and therefore, should be employed for the treatment or management of diabetes. Mechanistically the effect is due to the formation of adduct with carbonyl compounds thereby protecting the proteins from glycation (please refer Diagram 1 for details). Since, NAC is an FDA approved drug, it can be directly repurposed for the treatment of diabetes. Organosulfurs range from s-Allyl mercaptocysteine, SAMC; Diallyl trisulfide, DATS; Diallyl disulfide, DADS; Alliin, etc^65^. Further research should be conducted to identify most effective organosulfur with less side effects. Although, use of certain antibiotics has been reported to trigger diabetes^66,67^, organosulfur antibiotic like penicillin and sulfonilamide may also be employed as anti-diabetic pills. Of course, penicillin is currently used for the treatment of diabetic foot infections^66^. Recently organosulfurs have been advantageous for therapeutic intervention of several diseases including cardiovascular diseases^68^, cancer^69^ and neurodegenerative diseases^70^, etc. This study further expands the repertoire for the use of organosulfurs for the treatment of diabetes. The study also indicates that use of garlic could be beneficial for the treatment of diabetes. Despite of having advantages of using garlic or organosulfurs there are also associated risk factors. For instance, high garlic intake causes allergies, develop respiratory dysfunction like asthma, cough and even gastrointestinal troubles^71–73^. Study also reported that prolonged use of organosulfurs also develop adverse distress including disturbed metabolism and increased inflammatory response.

### Supplementary Information

Supplementary Figures.

## Data Availability

All data generated or analyzed during this study are included in this published article.
