# Supplementary material for: Organosulfurs, S-allyl cysteine and N-acetyl cysteine sequester di-carbonyls and reduces carbonyl stress in HT22 cells
Source: Sci Rep. 2023 Aug 11;13:13071. doi: 10.1038/s41598-023-40291-6 (PMC10421908; doi:10.1038/s41598-023-40291-6)
Supplement: Supplementary file 1 — Supplementary Figures. [file 41598_2023_40291_MOESM1_ESM.docx]

**Supplementary File**

**Organosulfurs, S-allyl cysteine and N-acetyl cysteine sequester di-carbonyls and reduces carbonyl stress in HT22 cells.**

Reshmee Bhattacharya^1^, Saakshi Saini^2^, Souvik Ghosh^2^, Partha Roy^2^, Nemat Ali^3^, Mohammad Khalid Parvez^3^, Mohammed S. Al-Dosari^3^, Awdhesh Kumar Mishra^4^ and Laishram Rajendrakumar Singh^1*^

^1^Dr. B. R. Ambedkar Center for Biomedical Research, University of Delhi, Delhi-110007.

^2^Department of Biosciences and Bioengineering, IIT Roorkee, Uttarakhand-247667.

^3^Depatment of Pharmacology and Toxicology, College of Pharmacy, King Saud University, Riyadh-11451, Saudi Arabia.

^4^Department of Biotechnology, Yeungnam University, Gyeongsan, Gyeongsanbuk-do, Republic of Korea.

^*^Correspondence author:

[lairksingh@gmail.com](mailto:lairksingh@gmail.com) (Prof. Laishram Rajendrakumar Singh), Mobile: +91-9811630757

[awdhesh](mailto:ywacko@catholic.ac.kr)[@ynu.ac.kr](mailto:lairksingh@gmail.com) (Prof. Awdhesh Kumar Mishra).

**Figure legends:**

**Figure S1. Profile of glycation of CA in different time frames.** Total carbonyl content generated from the covalent modification of CA by MGO. The concentration of CA used was 30μM.

**Figure S2. Activity status of MGO-modified CA in the absence and presence of SAC.** Dose-dependent measurement of CA activity in presence of different concentrations of SAC (0-2mM).

**Figure S3. Effect of SAC on the prevention of structural alteration in CA by MGO.** Representative tryptophan fluorescence spectra (A) and ANS fluorescence spectra (B) in the presence of MGO and MGO-SAC mixtures.

**Figure S4. Size distribution by volume of CA modified with MGO at different time frames.** (A-B), (C-D), (E-F), (G-H), (I-J), (K-L), (M-N) represents samples at Day 1, day 2, day 3, day 4, day 5, day 6, day 7 respectively. Left panels represent unmodified controls and right panels represent MGO-modified CA.

**Figure S5.** Relation between increase in AGE-specific fluorescence versus MGO concentration.

**Figure S6. Quantification of ROS levels.** Graph represents relative ROS levels of HT22 cells in the presence of MGO-BSA, and MGO-BSA-SAC mixtures.

**Figure S7. Quantification of apoptotic status** **in presence of MGO-BSA-SAC.**  Percent apoptotic cells in the presence of MGO-BSA, and MGO-BSA-SAC mixtures.

**Figure S8. Activity status of MGO-modified CA in the absence and presence of NAC.** Dose-dependent measurement of CA activity in presence of different concentrations of NAC (0-2mM).

**Figure S9. Quantification of ROS levels in presence of MGO-BSA-NAC mixtures.** Relative ROS levels of HT22 cells in the presence of MGO-BSA, and MGO-BSA-NAC mixtures.

**Figure S10. Quantification of apoptotic status in presence of MGO-BSA-NAC.** Percent apoptotic cells in the presence of MGO-BSA, and MGO-BSA-NAC mixtures.

**Figure S11. Glycation kinetics profile of CA in presence of SAC.** Time-dependent DLS measurements of MGO-modified CA in absence and presence of SAC (1mM).

**Diagram 1. Diagram representing mechanism of action of SAC/NAC against MGO-induced covalent modification of proteins.**

**Figure S1**


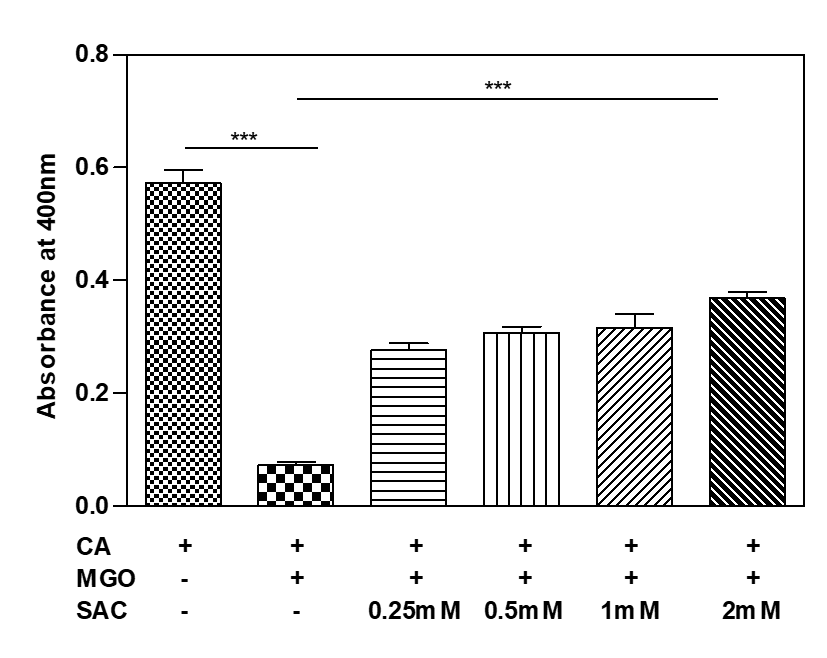


**Figure S2**

**Figure S3**


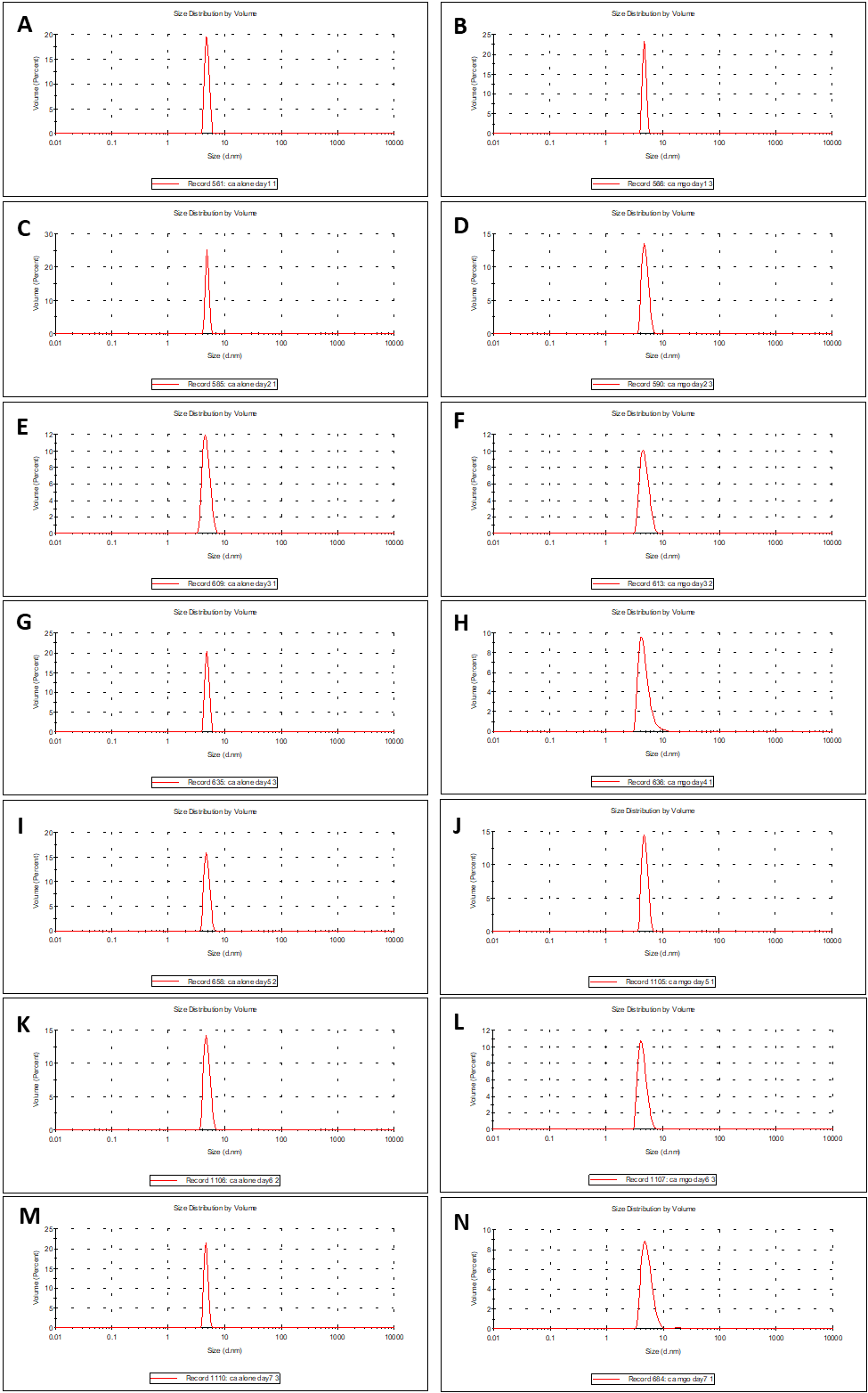


**Figure S4**

**Figure S5**


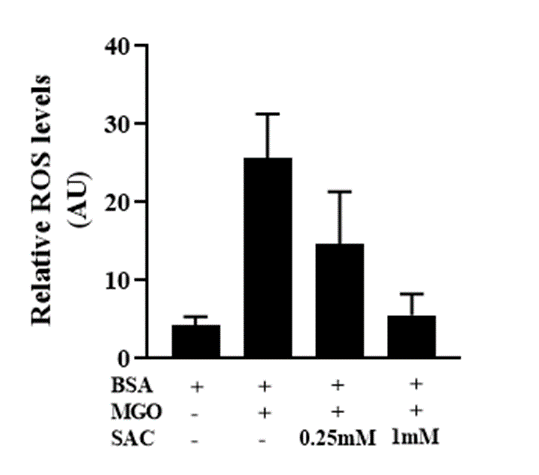


**Figure S6**

**Figure S7**

**
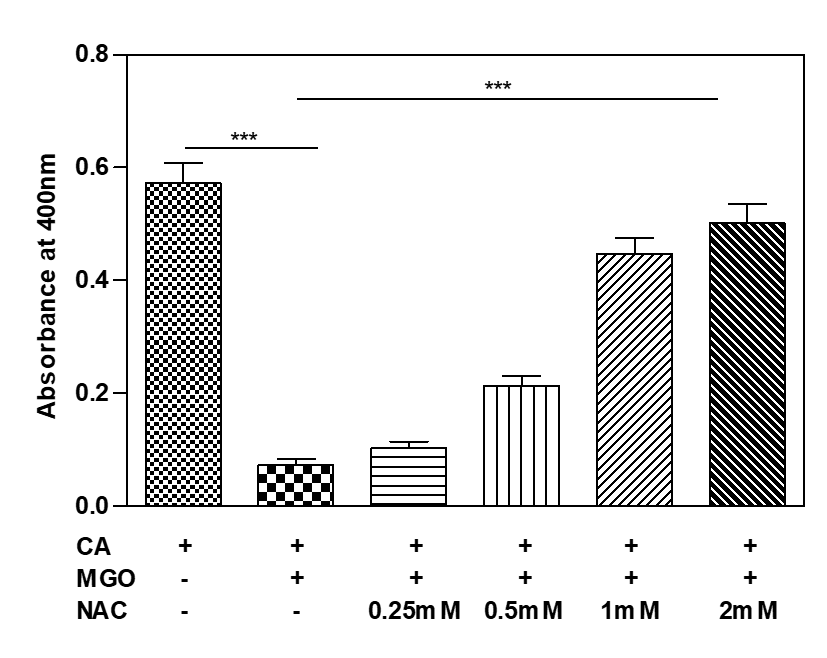
**

**Figure S8**


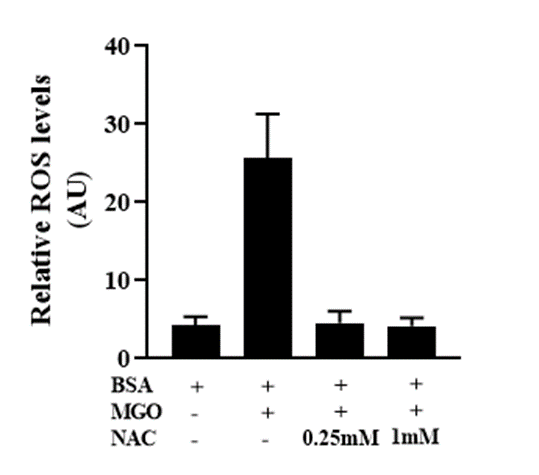


**Figure S9**

**Figure S10**

**Figure S11**


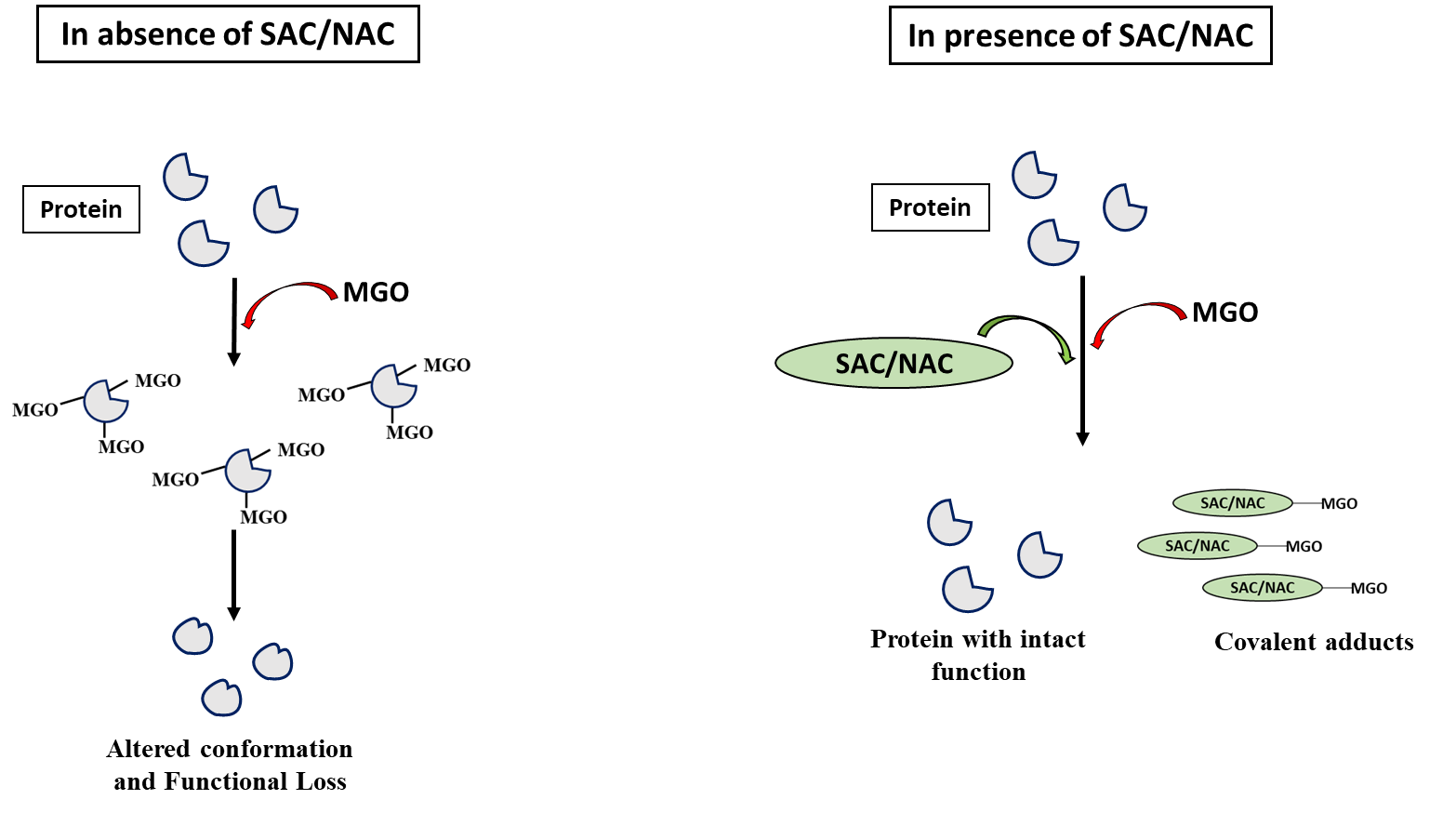


**Diagram 1**
